# Scattering of nanowire surface plasmons coupled to quantum dots with azimuthal angle difference

**DOI:** 10.1038/srep37766

**Published:** 2016-11-28

**Authors:** Po-Chen Kuo, Guang-Yin Chen, Yueh-Nan Chen

**Affiliations:** 1 Department of Physics, National Cheng-Kung University, Tainan 701, Taiwan; 2 Department of Physics, National Chung Hsing University, Taichung 402, Taiwan; 3 Physics Division, National Center for Theoretical Sciences, Hsinchu, Taiwan

## Abstract

Coherent scatterings of surface plasmons coupled to quantun dots have attracted great attention in plasmonics. Recently, an experiment has shown that the quantum dots located nearby a nanowire can be separated not only in distance, but also an angle *ϕ* along the cylindrical direction. Here, by using the real-space Hamiltonian and the transfer matrix method, we analytically obtain the transmission/reflection spectra of nanowire surface plasmons coupled to quantum dots with an azimuthal angle difference. We find that the scattering spectra can show completely different features due to different positions and azimuthal angles of the quantum dots. When additionally coupling a cavity to the dots, we obtain the Fano-like line shape in the transmission and reflection spectra due to the interference between the localized and delocalized modes.

Surface plasmons (SPs), or surface plasmon polaritons (SPPs), are propagating excitations of charge-density waves associated with the electromagnetic fields along the interface between a metal and a dielectric medium^1^,^2^,^3^,^4^,^5^,^6^. Surface plasmons in metallic nanostructures possess many advantages, such as enhanced transmission through subwavelength apertures^7^,^8^, amplification by stimulated emission of radiation^9^,^10^, enhanced photoluminescence from quantum wells^11^, enhanced fluorescence^12^,^13^,^14^,^15^, and surface-enhanced Raman scattering^12^,^16^. Moreover, there are many potential applications such as subwavelength imaging^1^,^17^,^18^, waveguiding devices below the diffraction limit^19^,^20^, biosensing^21^, and biological detection^22^. Therefore, designing and fabricating subwavelength optical devices using SPs^23^ open up new horizons of the research in this field.

With the tunable luminescence properties, such as localized surface plasmon resonances (LSPRs)^24^, plasmon-induced fluorescence enhancement^15^, broad excitation spectra, narrow emission spectra, and size-dependent emission^25^, quantum dot (QD) has recently attracted much attention for its ability to act as a photon detector^25^ or being an excellent single photon source^26^,^27^,^28^. On the other hand, metallic nanowire (MNW) is also an important class of plasmonic nanostructure for the SPs^29^,^30^,^31^,^32^,^33^,^34^, resonators^30^, sub-diffraction limit plasmon wave^31^, and plasmon lasers^10^.

Owing to the numerous advantages of both QD and MNW, QD that couples to MNW has emerged as an appealing system for coherent single-photon transport^35^ and long-range energy transfer with a high efficiency^36^. By the virtue of coherent transport, there are many extended applications, such as transistors^37^, plasmonic nanolaser^38^, quantum switch^39^,^40^, single-photon source^41^, biological sensing^42^,^43^, and nanoantennas^44^,^45^. Furthermore, the hybrid systems with exciton-plasmon interaction can reveal the features of cavity quantum electrodynamics^46^,^47^,^48^,^49^,^50^,^51^ and have applications in quantum information processing^5^,^52^,^53^,^54^,^55^.

A variety of experimental^33^,^36^,^41^,^56^,^57^,^58^,^59^,^60^,^61^,^62^ and theoretical works^9^,^35^,^39^,^40^,^42^,^47^,^48^,^49^,^50^,^51^,^63^,^64^,^65^,^66^,^67^,^68^,^69^,^70^,^71^,^72^,^73^,^74^,^75^,^76^,^77^ have been focused on the photon transport properties in the NW-QD systems. Recently, an experiment has reported that two QDs located nearby the NW are separated not only with a distance *d*, but also with an angle *ϕ* along the azimuthal direction^60^. Therefore, the difference in the angles between the QDs should be taken into account when investigating the scattering properities^33^,^34^,^48^,^78^.

In this work, we study the scattering spectra of the nanowire SPs coupled to double QDs with an azimuthal angle difference. We also consider the system comprising *N* QDs. Taking into account the angle difference between the dots, we study the scattrering properties of the SPs by using the transfer matrices. Compared to the double-dot case, we find the transmission/reflection profile reveals the periodic behavior for the three-dot case^58^ when rotating each QD along 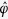 direction. We further study the scattering spectra of the Hybrid Quantum System (HQS) consisting of QDs and a metal nanoparticle^79^,^80^,^81^. It can be viewed as a cavity^82^,^83^,^84^,^85^ coupled to the NW-QD system. We find that the spectra reveal sharp and asymmetric response line shapes in the hybrid configuration. We analyze the results and provide explanations for the appearance of the Fano resonance.

## Results

### The Model

**Figure 1:**
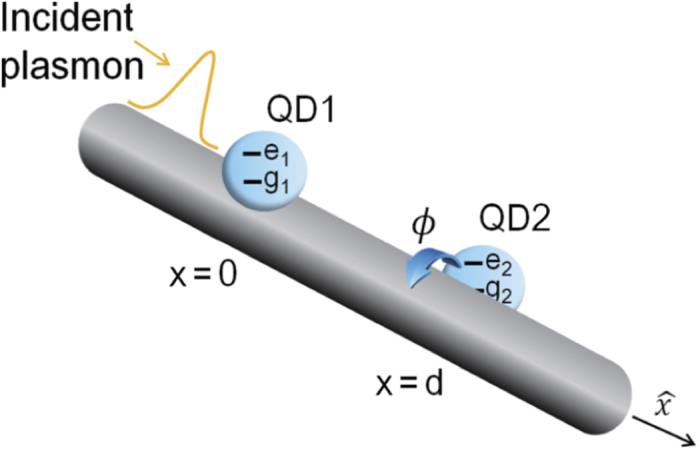
Schematic diagram of the two-QD model. Schematic diagram of the model: a metal nanowire coupled to two semiconductor QDs. A single surface plasmon injected from the left can be scattered by the two QDs which are placed on top of the nanowire with a inter-dot distance *d* along the 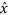 direction and an azimuthal angle *ϕ* along the 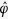 direction.

Lets us consider two identical QDs near a cylindrical metal nanowire. Assuming that they have the same separation from the metal wire, both with energy spacing *ħω*_0_, separated not only with a distance *d*, but also with an angle *ϕ* as shown in Fig. 1. Since the propagating modes are along the 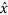 and 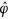 directions, the phase differences acquired by the second dot are *ikx* and *inϕ*, where *k* and *n* are the wave number and quantum number governing the *x* and *φ* components, respectively. Under the rotating wave approximation, the interactions between the propagating photons and quantum dots can be described by the Hamiltonian,


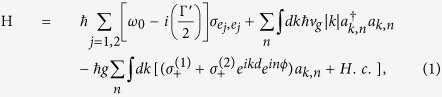


where 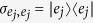 represents the diagonal element of the *j*^*th*^ QD operator with a atomic resonance frequency *ω*_0_ and 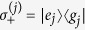 represents the rasing operator. Here, 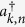 (*a*_*k*,*n*_) is the creation (annihilation) operator of the SP. We assume a SP is incident from the left with energy *E*_*k*_ = *v*_*g*_*k* for the *n*^*th*^ mode. Here, *v*_*g*_ and *k* are the group velocity and wave number of the incident SP, respectively. Since the SPs are confined on the surface of the cylindrical nanowire, the summation of *n* in Eq. (1) stands for the contributions from all the possible *n* modes, and *g* is the coupling constant between the SP and QD exciton. Note that Γ′ ≡ *γ*_0_ + Γ_0_ is the total dissipation including the decay rate into free space *γ*_0_ and other dissipative channels Γ_0_. By using the Fourier transform, each term in Eq. (1) can be easily represented in real space


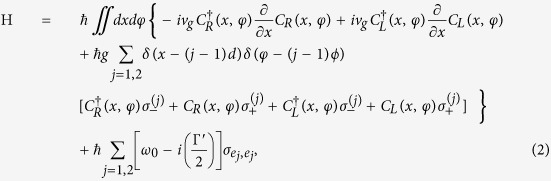


where 
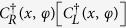
 is a bosonic operator creating a right-going (left-going) SP at *x* and *φ*. The stationary state of the above QDs-NW coupled system with the energy matching condition *E*_*k*_ = *v*_*g*_*k* can be written as


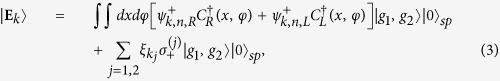


where |*g*_1_, *g*_2_〉 |0〉_*sp*_ denotes that both the QDs are in their ground states with zero SP state, and 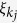 is the probability amplitude that the *j*^th^ QD jumps to its excited state. Suppose that a SP is incident from the left, the scattering amplitudes 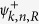 and 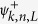 take the forms


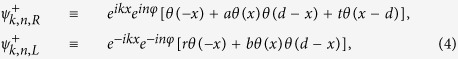


where *t* and *r* are the transmission and reflection amplitude, respectively. Here, *a* and *b* represent the probability amplitudes of the SP between *x* = 0 and *d, φ* = 0 and *ϕ*, respectively. Besides, *θ*(*x*) is the unit step function. From the eigenvalue equation, *H*|**E**_*k*_〉 = *E*_*k*_|**E**_*k*_〉, one can obtain the following relations for the coefficients:


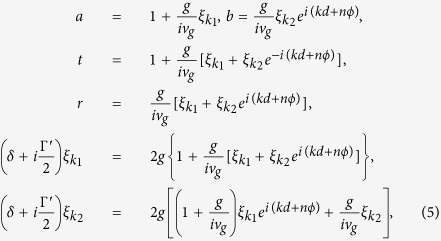


where 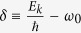 is the detuning between the incident SP energy with *E*_*k*_ and the QD exciton energy *ω*_0_. By solving Eq. (5), the exact forms of the transmission and reflection amplitudes, *t* and *r*, are given by


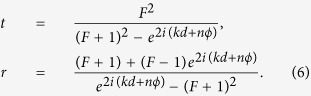


**Figure 2:**
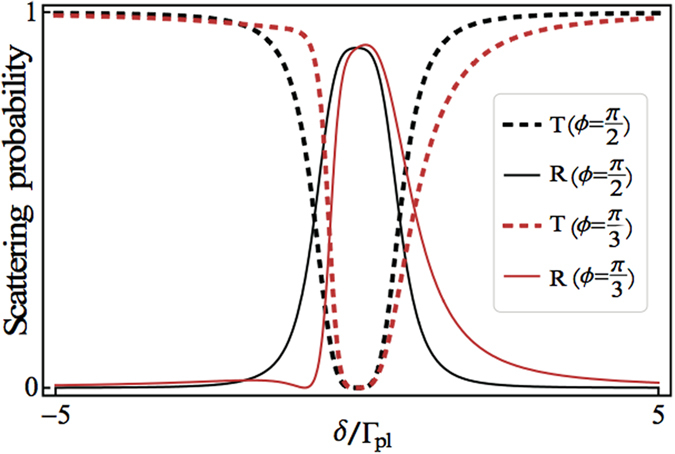
Effect from azimuthal angle difference in transmission spectra. The transmission probabilities |*t*|^2^ (dashed line) and reflection probabilities |*r*|^2^ (solid line) of the surface plasmon scattered by two QDs with azimuthal angle diference as a function of detuning 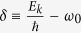. When *d* = 0 (here, it means the inter-dot distance is very small compared with the wavelength of incident fields), the two QDs are assumed to be at the same position.

Here, we have defined the function 
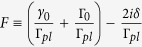
, where 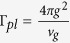 is the decay rate into the SP modes. The transmission and reflection probabilities of the SP are defined as *T* = |*t*|^2^ and *R* = |*r*|^2^, respectively, as shown in Fig. 2.

### Plasmon Scattered By N Quantum Dots

We now consider further a general model consisting of N identical QDs coupled to the SP. Under the rotating wave approximation, the interaction Hamiltonian becomes


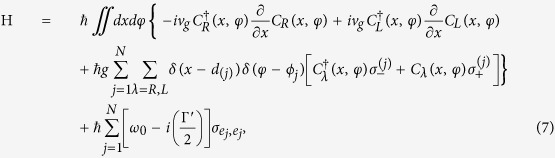


where *d*_(*j*)_ is the distance between the first dot and *j*^*th*^ dot, and *ϕ*_*j*_ is the angle of *j*^*th*^ QD with respect to the first QD along the 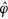 direction when setting *d*_1_ and *ϕ*_1_ being zero. On the other hand, the scattering property of a nanowire coupled to N identical QDs can also be studied by applying the transfer-matrix method. Let us briefly review the transmission amplitude *t* and the reflection amplitude *r* for the case of a single-dot coupled to the nanowire:


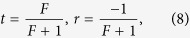


where *F* has been defined in Sec. II. By making use of the transmission and reflection coefficients in Eq. (8), the transfer matrix *T*_*q*_ of the NW coupled to a single-QD can be written as


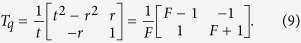


Thus, the transfer matrix *τ* for the entire system is determined by


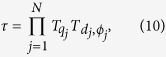


where


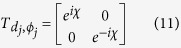


represents the transfer matrix of free propagation with 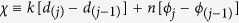. Consequently, the total reflect and transmit amplitudes with N QDs can be obtained:


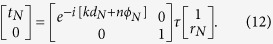


**Figure 3:**
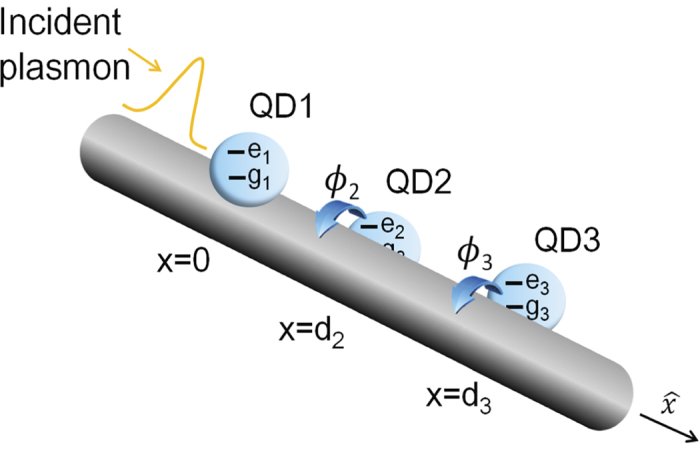
Schematic diagram of the three-QD model. Schematic diagram of the three QDs coupled to a metal nanowire with azimuthal angle difference along the 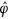 direction.

**Figure 4:**
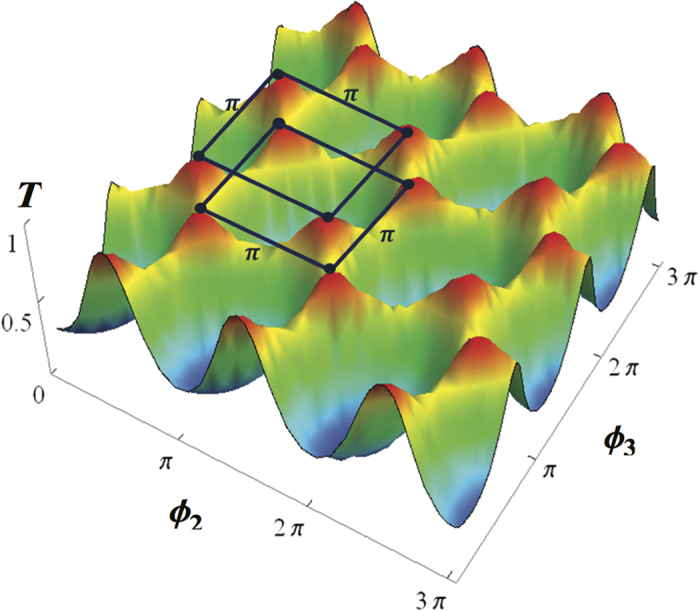
The interference of the azimuthal differences for the three-QD case. Analysis of the scattering probabilities of three quantum dots with the azimuthal angle differences between the second QD (*ϕ*_2_) and third QD (*ϕ*_3_) for the mode *n* = 1, *k* = 1, *d*_2_ = *π, d*_3_ = 2*π* and *δ*/Γ_*pl*_ = 1. Here, the red regions indicate the high transmission probability around unity.

In order to make a comparison to the double-dot case, we specifically consider the three-dot case^58^ as shown in Fig. 3. By solving the eigenvalue equation with *N* = 3 in Eq. (7) or Eq. (12), the transmission and reflection amplitudes can be obtained. For simplicity, we only show the transmission amplitudes


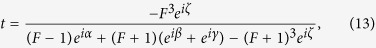


where we have defined the phase terms *ζ* ≡ 2*nϕ*_2_, *α* ≡ 2[*kd*_3_ + *n*(*ϕ*_2_ + *ϕ*_3_)], *β* ≡ 2(*kd*_2_ + 2*nϕ*_2_) and *γ* ≡ 2[*k*(*d*_3_ − *d*_2_) + *nϕ*_3_], respectively. Here, we are interested in the scattering spectra resulting from the varying angles of QD-2 and QD-3. Figure 4 shows the scattering spectra as functions of the angles *ϕ*_2_ and *ϕ*_3_. We find that the transmission (reflection) coefficient reveals the periodic maximum (minimum) value 1 (0), when keeping one QD fixed at the certain angle along the 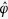 direction.

### QDs-NW System Coupled To Cavity

**Figure 5:**
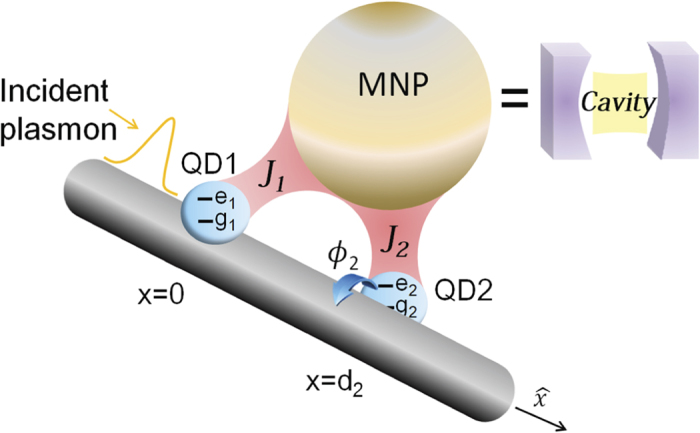
Schematic diagram of the QD-wire-nanoparticle hybrid system. Schematic diagram of the hybrid quantum system comprising QDs-wire and metal nanoparticle. The coupling strengths between the metal nanoparticle and QD-1, QD-2 are *J*_1_ and *J*_2_, respectively. The metal nanoparticle can be viewed as a special “cavity” in the strong coupling regime.

**Figure 6:**
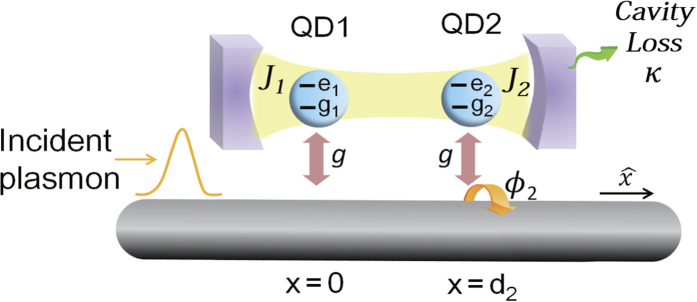
Schematic sketch of the effective hybrid model: common cavity. Schematic sketch of the effective hybrid model. Both QD-1 and QD-2 are coupled to a common cavity with a loss rate *κ* of the cavity photons. The transmission spectrum shows the Fano lineshape due to the interference between different channels. Here, the coupling strengths between cavity and QD-1, QD-2 are *J*_1_ and *J*_2_, respectively.

Recently, hybrid quantum system (HQS) has attracted renewed attention for its prospect of applications in future quantum devices. Here, we consider the HQS of the QDs (with nanowire) coupled to a metal-nanoparticle (MNP) as shown in Fig. 5. it was reported^82^,^83^,^84^,^85^,^86^,^87^ that, for the very small separation between a quantum emitter and a metal nanoparticle, the spectral density of the surface electromagnetic fields of the nanoparticle becomes Lorentzian. This indicates that the emitter-nanoparticle system can form an effective cavity quantum electrodynamics (QED) system. We therefore study the scattering spectra of two kinds of HQS comprising the cavity coupled to two QDs. For the first case, we assume both QD-1 and QD-2 are coupled to the same cavity as shown in Fig. 6. In real space, the Hamiltonian of the cavity photon with a loss rate *κ* can be written as


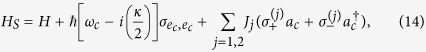


where 
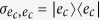
 is the diagonal element of the cavity operator, and 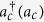 is the bosonic creation(annihilation) operator of the cavity mode. Here, *J*_*j*_ represents the coupling strength between the cavity and *j*^*th*^ QD. The transmission and reflection coefficients can be written as


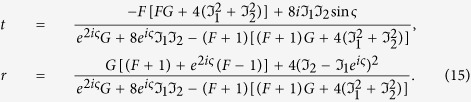


**Figure 7:**
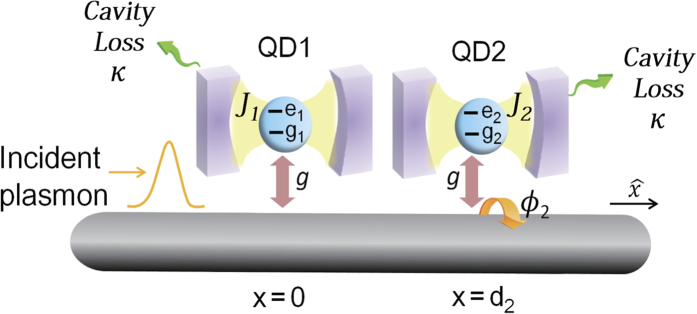
Schematic sketch of the effective hybrid model: individual cavity. Schematic draw of the model for each QD is individually coupled to different cavities with the same loss rate *κ* of the cavity photons.

Here, we have defined the function 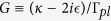, 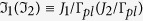, and the phase term 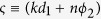. The detuning between the incident SP energy (with *E*_*k*_) and the cavity resonant frequency (*ω*_*c*_) is labeled by the symbol *ε*. For the second case, we study the configuration that each QD is individually coupled to its own cavity as shown in Fig. 7. Here, we have assumed the two cavities are identical for simplicity. The Hamiltonian of the composite system can be rewritten as


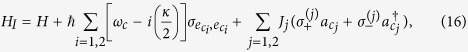


where 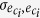 represents the diagonal element of the *i*^*th*^ cavity operator, and 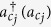 is the bosonic creation(annihilation) operator of the *j*^*th*^ cavity mode. Also, the scattering coefficients can be obtained by solving eigenvalue equation:


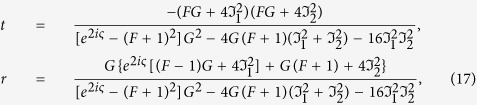


**Figure 8:**
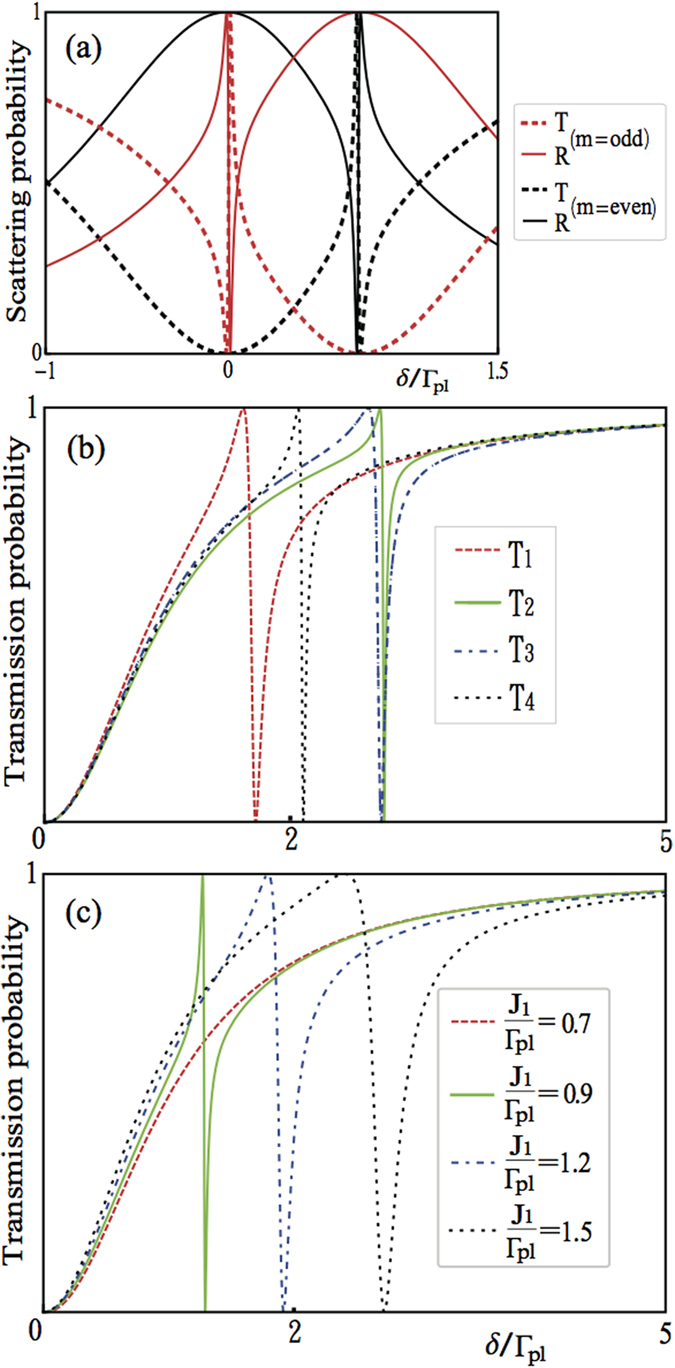
Scattering spectra for the two QDs coupled to common nanoparticle. (**a**) The transmission (dashed) and reflection (solid) probabilities as a function of detuning 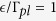, 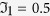 and 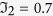 when *kd*_1_ + *nϕ*_2_ = *mπ* with *m* being an odd (red) or even (black) integer. (**b**) When *m* is an odd integer and 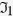 is close to 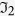 with appropriate value of *ϵ*/Γ_*pl*_, the typical transmission spectra present more distinct Fano resonance for the first case. For Γ′ = 0 and *κ* = 0, we plot the transmission probabilities *T*_1_(red-dashed), *T*_2_(green-solid), *T*_3_(blue-dot-dashed), *T*_4_(black-dotted) with the detuning *ϵ*/Γ_*pl*_ = 0.05, 0.6, 1.2, 3 when 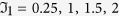, 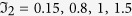, respectively. (**c**) The transmission probabilities T as a function of *ϵ*/Γ_*pl*_ = 1, 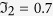 for Γ′ = 0 and *κ* = 0 when 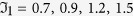, respectively.

**Figure 9:**
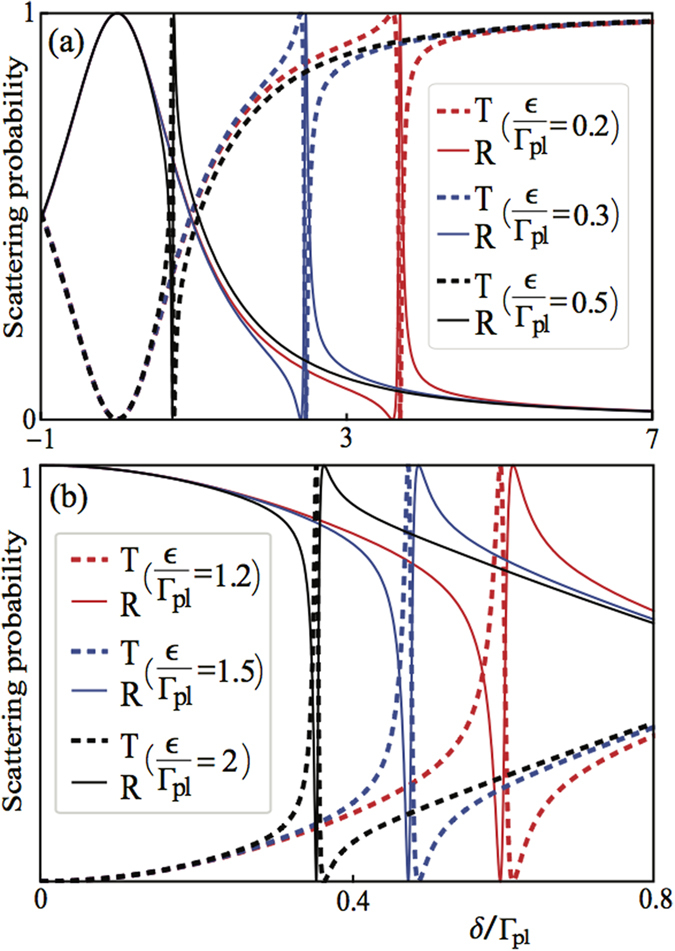
Analysis of Fano resonance. The transmission (dashed) and reflection (solid) probabilities as a function of 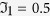, 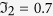 for Γ′ = 0 and *κ* = 0 when (**a**) *ϵ*/Γ_*pl*_ = 0.2, 0.3, 0.5, (**b**) *ϵ*/Γ_*pl*_ = 1.2, 1.5, 2, respectively. The position of the Fano lineshape can be shifted from the right to the left along the *δ*/Γ_*pl*_ axis when increasing the detuning *ϵ*/Γ_*pl*_.

**Figure 10:**
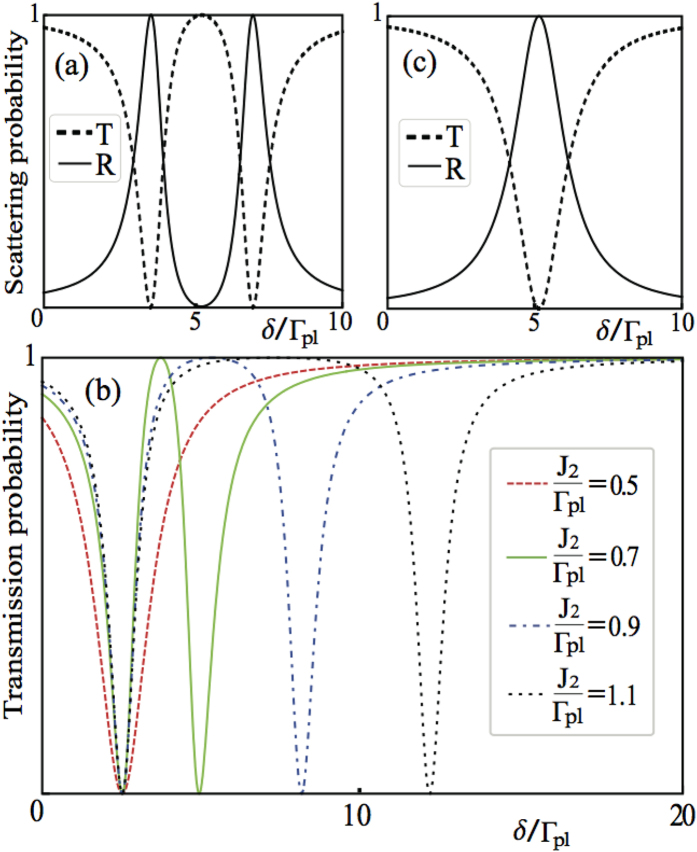
Scattering spectra for the two QDs coupled to individual nanoparticles. For the second case, we set 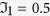, Γ′ = *κ* = 0, and *kd*_1_ + *nϕ*_2_ = *mπ* with *m* being an integer. (**a**) The transmission (dashed) and reflection (solid) probabilities as a function of 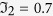 and *ϵ*/Γ_*pl*_ = 0.07. This panel shows a standard Breit-Wigner lineshape without the Fano resonance for two QDs. (**b**) The transmission probabilities as a function for 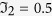 (red-dashed), 0.7 (green-solid), 0.9 (blue-dot-dashed), 1.1 (black-dotted), respectively with *ϵ*/Γ_*pl*_ = 0.1. By adjusting coupling strength between each QD to the cavity, the position of each peak along the *δ*/Γ_*pl*_ axis can be controlled. (**c**) When 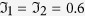, the overlap of two peaks makes the two QDs act like a single QD in transmission(dashed) and reflection(solid) probabilities with *ϵ*/Γ_*pl*_ = 0.07.

We plot in Figs 8, 9 and 10 the transmission probabilities *T* = |*t*|^2^ (dashed lines) and reflection probabilities *R* = |*r*|^2^ (solid lines) as a function of the detuning for the both cases. In plotting Fig. 8, we find that when *kd*_1_ + *nϕ*_2_ = *πm* with *m* being an integer and 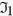 being close to 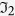 with appropriate value of *ϵ*/Γ_*pl*_, the transmission and reflection spectra have a more distinct Fano-type line shapes. In Fig. 9, when increasing the detuning *ϵ*/Γ_*pl*_, the position of the Fano-type line shapes would be shifted from the right to the left along the *δ*/Γ_*pl*_ axis. For the second case, however, we can only observe two peaks with the absence of asymmetric Fano-type line shape as shown in Fig. 10. When increasing the detuning *ϵ*, the inter-peak separation is reduced rapidly.

## Discussion

Since the Fano resonance only occurs in the first case, it is interesting to ask: What makes the two cases different? To answer this, let us note that, in Fig. 8(b), the stronger coupling strength of the two QDs to the cavity, the larger detuning *ϵ* is required to form the Fano-type line shapes for the first case. Contrarily, when *J*_1_ coincides with *J*_2_, the Fano resonance vanishes rapidly. In this regard, the Fano resonance arises from the constructive and destructive interference between the localized and delocalized channels by the virtue of the coupling of the two QDs to the same cavity. Here, the localized channel represents the single QD mode, and the delocalized channel denotes the hybridization mode of the cavity photon and the two dots^88^. The surface plasmons passing through the two channels carry different phases and result in the interference. On the other hand, we can easily control the position of each peak along the *δ*/Γ_*pl*_ axis by adjusting the coupling strength between each QD to the cavity in the second case as shown in Fig. 10. When *J*_1_ = *J*_2_, the overlapping of two peaks makes the two QDs collectively act like a single QD. The notable feature of these results indicates that the Fano-type line shape can’t be created due to the individual coupling to each own cavity. In other words, the difference between *J*_1_ and *J*_2_ is the primary cause of the Fano resonance.

In conclusion, the real-space Hamiltonians and transfer-matrix method are used to obtain the transport properties of SPs propagating on the surface of a silver NW coupled to QDs. The transmission and reflection spectra of the SPs depend not only on the position, but also on the azimuthal angle of the QDs. For the double-dot case, even the two QDs are placed at the same position in the 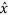-axis, changing the angle of a QD along 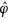 direction also affects the reflection (transmission) spectra. For the triple-dot case, the transmission (reflection) coefficient reveals the periodic maximum (minimum) value when keeping one QD fixed at the certain angle along the 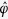 direction. Moreover, when there is an additional cavity coupled to QDs, the Fano-type line shape can be created if both the QDs are coupled to the same cavity. The appearance of Fano resonances is attributed to the interference between the localized and delocalized modes.

## Additional Information

**How to cite this article**: Kuo, P.-C. *et al*. Scattering of nanowire surface plasmons coupled to quantum dots with azimuthal angle difference. *Sci. Rep.* **6**, 37766; doi: 10.1038/srep37766 (2016).

**Publisher's note:** Springer Nature remains neutral with regard to jurisdictional claims in published maps and institutional affiliations.
